# Hospital readmission within 10 years post stroke: frequency, type and timing

**DOI:** 10.1186/s12883-017-0897-z

**Published:** 2017-06-19

**Authors:** Gitta Rohweder, Øyvind Salvesen, Hanne Ellekjær, Bent Indredavik

**Affiliations:** 1 From the Stroke Unit, Department of Internal Medicine, St Olav’s Hospital, University Hospital of Trondheim, Harald Hardraades gate 5, 7030 Trondheim, Norway; 2 The Institute for Neuromedicine (INM), Faculty of Medicine and Health Sciences, Norwegian University of Science And Technology (NTNU), Trondheim, Norway; 3 The Unit of Applied Clinical Research, Faculty of Medicine and Health Sciences, Norwegian University of Science And Technology (NTNU), Trondheim, Norway

**Keywords:** Stroke, Readmission, Readmission rate, Longterm follow-up, Secondary prophylaxis

## Abstract

**Background:**

The aim of this study was to examine the hospital readmissions in a 10 year follow-up of a stroke cohort previously studied for acute and subacute complications and to focus on their frequency, their causes and their timing.

**Methods:**

The hospital records of 243 patients, 50% of a cohort of 489 patients acutely and consecutively admitted to our stroke unit in 2002/3, were subjected to review 10 years after the incidental stroke and all acute admissions were examined. The main admitting diagnoses were attributed to one of 18 predefined categories of illness. Additionally, the occurrence of death was registered.

**Results:**

After 10 years 68.9% of patients had died and 72.4% had been readmitted to the hospital with a mean number of readmissions of 3.4 (+15.1 SD). 20% of the readmissions were due to a vascular cause, 17.3% were caused by infection, 9.3% by falls with (6.1%) and without fracture, 5.7% by a hemorrhagic event. The readmission rate was highest in the first 6 months post stroke with a rate of 116.2 admissions/100 live patient-years. Falls with fractures occurred maximally 3–5 years post stroke.

**Conclusions:**

Hospital readmissions over the 10 years following stroke are caused by vascular events, infections, falls and hemorrhagic events, where the first 6 months are a period of particular vulnerability. The magnitude and the spectrum of these long-term complications suggest the need for a more comprehensive approach to post stroke prophylaxis.

## Background

Hospital readmission within 30 days post discharge is used as a quality indicator for the delivery of medical care in the aftermath of an acute hospital admission for many admitting diagnoses, and also for a diagnosis of stroke [1, 2]. Hospital readmission in the long term post stroke should reflect the problems present in the chronic phase of the disease and give information about the following parameters: the patient’s functional disability, stroke-related complications, comorbidity, efficacy of secondary prevention, care aspects, as well as the aging process, as they affect the stroke patient.

One year post stroke, 40.4% of patients had been readmitted in a US Medicare study and only 15% of patients survived admission-free for 5 years. The most common causes for readmission were pneumonia, stroke, acute myocardial infarction, and congested heart failure [3]. In a one-year follow-up study using the Scottish NHS register, 15% of post-stroke readmissions were caused by infections, gastrointestinal complications and immobility-related problems [4].

The focus of post-stroke care has traditionally been secondary stroke prevention and the reduction in case fatality. For both, the last several decades have shown improvement with both short term and long term case fatality declining steadily since the 1950’s, in Europe and Scandinavia as well as the US [5–10]. The establishment of stroke units (SU) in Scandinavia and Central Europe may have contributed to the further reduction in measured stroke mortality since the year 2000 [11, 12]. In the original cohort randomized to our stroke unit we were able to document a 33% reduction in mortality, which persisted over the 10 years following the incidental stroke [11]. Gains have also been made in secondary stroke prevention and are likely a consequence of improved risk factor control including lifestyle changes (reduction in salt intake and smoking) and medical interventions [8]. Secondary cardiovascular prevention was named first as a goal of treatment in 1993 and has received increased attention since, the medical regimen being essentially equivalent to stroke prevention [5]. Other, nonvascular post-stroke morbidity is less defined in the stroke-literature and also not addressed with preventive measures. The aim of this study was to examine the readmissions and the case fatality in a 10 year follow-up of a stroke cohort acutely treated in a stroke unit and previously studied for acute and subacute complications [13, 14], and to focus on the frequency, causes and timing of the readmissions.

## Methods

The SU, Dept. of Medicine is located at the University Hospital of Trondheim, which serves as the primary hospital for the approximately 200,000 inhabitants of Southern Trondelag and as the tertiary hospital for Central Norway. A cohort of 489 patients, acutely and consecutively admitted to the SU in 2002/3, were prospectively screened for complications during the first week [13]. After discharge from the SU, all patients were followed up by an early supported discharge service (ESD), previously described, and received an outpatient follow-up evaluation 4 weeks after discharge with a focus on optimal secondary stroke prevention and screening for complications [15]. 50% of the patients were randomly allocated to be prospectively screened for complications by weekly telephone interview during the first 3 months post stroke. Patients were included regardless of discharge destination, and they received no therapeutic interventions in this context. The frequency and type of the complications and their association with functional outcome have previously been published [13, 14]. The hospital records of 243 of these 244 patients with follow-up data were subjected to a review after 10 years. One patient could not be re-identified and was, therefore, lost to follow-up. All acute admissions to the hospital following the incidental stroke admission were examined, the main admitting diagnosis was verified with the help of the discharge summary, and the admission was assigned to one of 18 predefined categories. Additionally, and as a procedural control, the International Classification of Diseases (ICD) 10 codes were logged and categorized. This secondary procedure was confirmatory. The predefined categories were: 1.Ischemic stroke 2.Hemorrhagic stroke 3.Cardiovascular and cardiac events 4.Peripheral vascular events 5.Chest infection 6.Serious infection 7.Urinary tract infection (UTI) 8.Falls with fractures 9.Falls without fractures 10.Epileptic seizures 11.Stroke sequelae 12.Hemorrhagic events (documented gastrointestinal bleeds, hemoptysis as well as iron deficiency anemia) 13.Venous thrombotic events 14.Renal insufficiency 15.Transient ischemic attack (TIA) 16.Cardiac angina 17.Cancer 18.Other.

### Data analysis

An event-file for hospital readmissions was established with readmissions to our hospital linked to the patients. As Trondelag has a stable patient population, as established by the HUNT-study [16], and the University Hospital is the only hospital in the district of Southern Trondelag, no other hospital needed to be contacted. The readmission dates were logged and we analysed for frequency of the categories and computed readmission rates (incidence per 100 live patient-years) for the following time intervals: 1 month, 3, 6, 12 (1), 36 (3), 60 (5), 84 (7) and 120 months (10 years). Subsequently, the previously established patient file was updated with each of the 243 patients’ date of death, if death had occurred according to the local register (PAS), which is linked to the National Registry (Folkeregistret). The cumulative incidence of readmissions among the patients alive could then be calculated. The data were analysed in IBM SPSS statistics version 22. The readmission rates per 100 live patient-years were obtained by using R – version 2.13.1 [17]. The rates were corrected for death as a competing event.

## Results

The patients’ baseline characteristics are presented in Table 1. 56% of the patients were female, the mean age was 76.5 years (SD 9.8). 90% had an ischemic stroke, 10% had a hemorrhagic stroke. The patients’ functional status was assessed with the modified Rankin scale (mRS) [18]. The mean estimated pre-stroke mRS was 1.6 (SD 1); the mean mRS day 1 was 3.5 (SD 1.3).

**Table 1:** Baseline Characteristics of the Patients (*n* = 243*)

Characteristics	N	%
Female sex	*137*	56
Age, Mean (SD)	76.5 (9.8)	
Risk Factors		
Hypertension	93	38
Previous stroke	47	19
Atrial fibrillation	46	19
Diabetes mellitus	30	12
Prev. myocardial infarction	35	14
Smoking	48	20
TIA	24	10
Diagnosis		
Ischemic stroke	218	90
Hemorrhagic stroke	25	10

*One patient could not be re-identified from the cohort of 244 and was, therefore, lost to follow up

Table 2 shows the proportion of patients alive and never readmitted, alive and readmitted, dead and readmitted, dead and never readmitted in the time-periods noted. All time intervals were calculated from the admitting-date of the incidental hospital admission. After 1 year, 23.5% of the patients were dead with the majority (17%) never readmitted to the hospital; 40.3% of patients had been readmitted to the hospital. At 5 years, 49.4% of the patients had died and 67.5% of all patients had been readmitted to the hospital. After 10 years, 68.9% of the patients had died and 72.4% had been readmitted to the hospital. This left 31.1% of the patients alive and 27.6% never readmitted after 10 years of follow-up.

**Table 2:** Cumulative Readmission or Death by Patients (*N* = 243)

	Alive		Alive		Dead		Dead	
	Never Readmitted		≥1 Readmissions		≥1 Readmissions		Never Readmitted	
Time Period	N	%	N	%	N	%	N	%
30 days	209	86.5	13	5.4	0	0	21	8.6
3 months	164	67.5	40	16.5	3	1.2	36	14.8
6 months	120	49.4	71	29.2	10	4.1	42	17.3
1 year	102	42.0	84	34.5	14	5.8	43	17.7
3 year	55	22.6	96	39.5	45	18.5	47	19.4
5 year	28	11.5	95	39.1	69	28.4	51	21.0
7 year	24	9.8	76	31.3	92	37.9	51	21.0
10 year	16	6.6	59	24.5	116	47.9	51	21.0

**Fig. 1:**
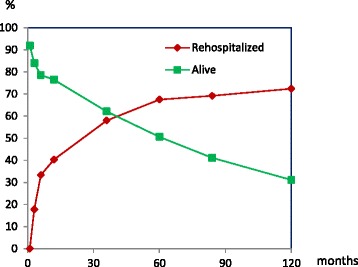
Cumulative Incidence: Percentage of Patients Alive vs. Patients Rehospitalized

Figure 1 displays the percentage of stroke patients readmitted and alive at the defined intervals of 1, 3, 6, 12 (1),36 (3), 60 (5), 84 (7) and 120 months (10 years). 831 acute hospital readmissions were identified in the medical records belonging to 72.4% of the patients (177/243). The mean number of readmissions per patient was 3.4 (SD 15.1); the median number was 2 (range 0–24).

The categories of the main diagnoses and the frequencies with which they caused admission to the hospital are displayed in Table 3. In summary, five categorical groups were responsible for readmissions: I) 20% of the readmissions were due to a vascular cause: ischemic stroke 5.4% + hemorrhagic stroke 1.1% + cardiovascular/cardiac event 10.5% + peripheral vascular event 3.0%. II) 17.3% of readmissions were caused by infections: chest infection 9.0% + serious (systemic) infection 4.1% + UTI 4.2%. III) Readmissions due to falls accounted for 9.3%: falls with fractures 6.1% + falls without fractures 3.2%. IV) Hemorrhagic events accounted for 5.7% V) Cancer 8.5%. The category ‘Other’ constituted 24.7% of the readmissions and consisted of many different diagnoses.

**Table 3:** Acute Hospital Readmissions According to Category of Illness

Category	N	%
Ischemic stroke	45	5.4
Hemorrhagic stroke	9	1.1
Cardiovasc. and cardiac events	87	10.5
Peripheral vasc. Events	25	3.0
Chest infection	75	9.0
Serious infection	34	4.1
UTI	35	4.2
Falls with fractures	51	6.1
Falls without fractures	27	3.2
Epileptic seizures	24	2.9
Stroke sequelae	28	3.4
Hemorrhagic events	47	5.7
Thrombotic events	10	1.2
Renal insufficiency	17	2.0
TIA	18	2.2
Cardiac angina	23	2.8
Cancer	71	8.5
Other	205	24.7
Total	831	100

**Fig. 2:**
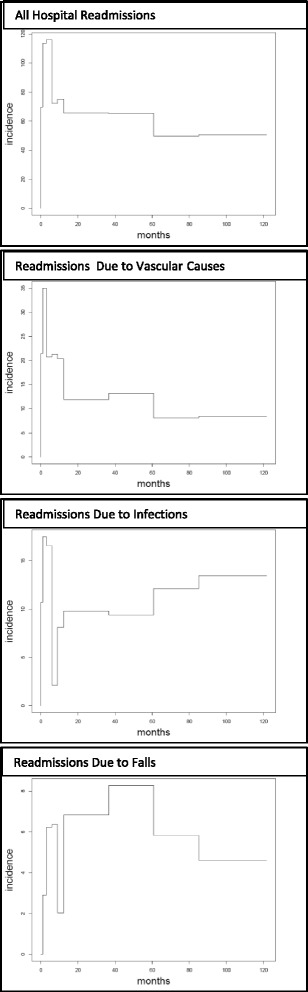
The Incidence of Hospital Readmissions per 100 Live Patient-Years

In Fig. 2 the readmission rates per 100 live patient-years are shown. These rates were calculated for all readmissions collectively, as well as separately for those due to vascular causes, infections and falls. Overall, readmissions reached a maximal rate of 116.2 admissions/100 live patient-years in the first 6 months post-stroke. Both, readmissions due to vascular causes and infections also had their peak rates in the first 6 months at 35.0 and 17.5 admissions/100 live patient-years respectively. The admission rate for falls was maximal between three and five years post-stroke at 8.3 admissions/100 live patient-years. After approximately 5 years, the readmission rate for all causes stabilized at 50.7 admissions/100 live patient-years, for vascular causes at 8.5 admissions/100 live patient-years and for falls at 4.6 admissions/100 live patient-years. Admissions due to infectious causes, however, increased steadily from 9.4 to 13.5 admissions per 100 live patient-years between five and ten years post-stroke.

## Discussion

Hospital readmission was a frequent event in our study, with 46% of readmitted patients presenting during the first 6 months, 56% during the first year and 94% during the first 5 years. The causes for readmission were heterogeneous and due to cardiac and vascular causes, infections, falls +/− fractures, hemorrhagic events and cancer of all forms. Cardiac and vascular causes was the largest category at 20% and included recurrent stroke at 6.5% of readmissions. Infections accounted for 17.3% of readmissions. While cancer is a stroke-unrelated event, falls, fractures and hemorrhage can be a consequence of the incident stroke, its sequelae or its treatment with antithrombotic medication. Of note are the relatively infrequent occurrences of post-apoplectic seizures and of venous thrombotic events.

Observed over time, the highest readmission rates occurred within the first 6 months post stroke, which must be regarded as a particularly unstable and vulnerable period of time. This was especially true for serious cardiac and vascular events requiring hospitalizations, which occurred at a rate of 30 per 100 live patient-years in the first 6 months and appeared to reach a steady-state after approximately 5 years post-stroke at 8.5 per 100 live patient/years. A similar rate of 8.9 nonfatal vascular events per 100 patient-years was observed at three-year follow-up in the international REACH-register of vascular disease [19]. The drop-off in readmission rates at approximately 5 years post stroke may well be due to the fact that the most seriously affected stroke patients died within this time period, as has been shown in a large cohort of the Swedish stroke register [20]. Admissions due to falls peaked between three and five years post stroke, and the majority of these were accompanied by a fracture. Infections had a continuous presence with a slight but steady increase in rate after 5 years.

How do the frequency and causes for readmission post stroke compare to the occurrences in the general elderly population? A US Medicare linkage study conducted in the year 2000 compared a cohort of 823 first ever stroke patients with a matched cohort (age, sex and race) of 4115 patients over 5 years [21]. The hospital readmission rates were doubled in the stroke cohort. The readmissions for ischemic stroke were 4 times as frequent in the stroke cohort, readmissions for heart failure, cardiac events, pneumonia and hip fracture twice as frequent in the stroke cohort as in the non-stroke cohort [21]. In Europe, no case-control study has been performed to our knowledge, but there are a few studies regarding general hospital admissions. A study of acute admissions to medical departments in Denmark from 2010 showed that 24% of hospital admissions in the age group 60+ were due to cardiac and vascular events, 17% due to infection [22]. A multi-center European study reported in 2009 that 23% of hospital-admissions were due to cardiac and vascular events, 15% due to infection in a population with the median age of 67 years [23]. The US study was able to document a much higher rate of readmissions in the stroke cohort versus the matched general population cohort over the first 5 years post-stroke. In contrast are our results after 10 years seemingly very similar to the distribution of readmissions in the general population. It may be that stroke patients have an increased vulnerability for the first 5 years post-stroke compared to the general population, and that readmissions approach a background level thereafter. Readmissions due to falls and fractures occur at a maximal rate 3–5 years post stroke in our study. Falls were also found to occur twice as frequently in a cohort of Norwegian patients studied 10 years post stroke than in the controls [24]. These fall-related events deserve further study in the stroke population and might justify increased efforts at prevention.

The post stroke case fatality lies at 23.5%, 49.4% and 68.9% at 1, 5 and 10 years respectively in our study. These numbers compare with 26.1% and 51.4% at 1 and 5 years in the US-study by Bravata et al. [3] published in 2007, and with 24.6%, 59.1% and 75.5% with the results from our stroke unit between 1986 and 1996 [11]. In a nationwide population-based cohort study using the Danish National Registry of Patients 5 year mortality decreased from 56.4% (1994–98) to 46.1% (2009–2011) for ischemic stroke and 66.1% to 61.0% for intracerebral hemorrhage [25]. These numbers document a significant decrease in 10-year case fatality over the course of the last 20 years. However, they also demonstrate that approximately half the stroke population continues to meet death within 5 years of the incidental stroke.

In the primary care literature, patients with stroke are recognized as one of several patient groups characterized by multiple chronic conditions, which show a greater hospitalization rate and a greater complication rate per hospitalization, compared with a patient group without these chronic conditions [26]. These patients, characterized by ambulatory care sensitive conditions, are considered a target for prospective ambulatory management [27, 28]. The interventions are rather nonspecific and consist of home-based care to avoid medication errors, volume depletion and fluid overload. In the post-stroke patients, these nonspecific interventions might be especially beneficial during the first 6 months. This could be a new task for early supported discharge teams.

Initiating specific measures to reduce chest-infection and fractures could reduce the burden of complications and their corresponding rehospitalizations. Measures to reduce chest infection include dysphagia protocols, adjustment of oral intake, immunization against influenza and Streptococcal infection. Measures to reduce fractures include fall prevention and could also, and perhaps more importantly, include assessment of osteoporosis and initiation of prophylactic treatment, perhaps at the time of the incident stroke. General measures to insure optimal nutrition and to encourage physical exercise will contribute to the reduction of infection, bone loss and physical frailty.

This observational study is limited by its small sample size and the lack of a non-stroke cohort. Its foremost strength is that it was conducted thoroughly, including chart reviews and validation of diagnoses for each patient.

Long-term follow-up of stroke patients is most efficiently achieved in the context of stroke registers, where one can gather an overview of stroke care on a macroscopic level. Patient-centered outcome measures are being developed for follow–up of stroke patients. The parameters chosen for both need to be clinically relevant [29]. However, there are few clinical studies that outline and analyze stroke outcomes in the long term and can help us determine long-term relevance [30]. With this study we hope to have contributed to this body of evidence.

## Conclusions

Hospital readmissions over the 10 years following stroke are caused by vascular events, infections, falls and hemorrhagic events, where the first 6 months post stroke are a period of special vulnerability. The magnitude and the spectrum of these long-term complications call for a reevaluation and a more comprehensive approach to post stroke prophylaxis.

## Abbreviations

ESD: Early supported discharge
ICD: International classification of diseases
mRS: Modified Rankin Scale
SU: Stroke unit
TIA: Transient ischemic attack
UTI: Urinary tract infection
